# Laparoscopic retroperitoneal resection of the duodenal gastrointestinal stromal tumors in neurofibromatosis type 1; Case Report and literature review

**DOI:** 10.3389/fsurg.2022.939705

**Published:** 2022-08-26

**Authors:** Al-Danakh Abdullah, Safi Mohammed, Mohammed Alradhi, Xinqing Zhu, Deyong Yang

**Affiliations:** ^1^ Department of Urology, First Affiliated Hospital of Dalian Medical University, Dalian, China; ^2^ Department of Respiratory Diseases, Shandong Second Provincial General Hospital, Shandong University, Jinan, China; ^3^ Department of Urology, The Affiliated Hospital of Qingdao Binhai Univesity, Qingdao, China; ^4^ Department of Surgery, Healing Hands Clinic, Dalian, China

**Keywords:** neurofibromatosis, gastrointestinal stromal tumors, retroperitoneal tumor, retroperitoneal approach, laparoscopic resection

## Abstract

**Background:**

Neurofibromatosis type 1, also known as NF1, is a disorder that is passed down in an autosomal dominant manner. It manifests in a wide variety of tumors and affects several organ systems. It is expected that those carrying the NF1 gene will develop a rare mesenchymal tumor known as a gastrointestinal stromal tumor (GIST) more than general population.

**Case report:**

This research discusses a 42-year-old female patient with NF1 who was identified with a duodenal GIST but clinically and radiographically misinterpreted as having a retroperitoneal neurofibroma. She had minimally invasive retroperitoneal laparoscopic surgery to remove the tumor and primary anastomosis of the affected duodenal wall. A spindle cell GIST was entirely excised during surgery, as indicated by the pathologist. As a consequence of dialogue at a multidisciplinary team meeting, the patient was discharged from the hospital on the fourth postoperative day and is presently undergoing regular clinical follow-up.

**Conclusion:**

Anatomically problematic sites, such as the duodenal GIST in NF1 patients, can be treated safely with the laparoscopic retroperitoneal approach even when retroperitoneal neoplasia arises from the intrabdominal structure and protrudes into the retroperitoneal region.

## Introduction

Neurofibromatosis type 1 (NF1), also known as von Recklinghausen's disease, is an inherited autosomal dominant syndrome that impacts many body organ systems and manifests clinically in various ways (1). NF1 is the most prevalent of the three neurofibromatoses, with a birth incidence of 1 in 2000, which is characterized by neurofibromas (peripheral nerve tumors) that cause skin abnormalities and bone deformation (2). In contrast, schwannomatosis (SWN) and NF2 are rare, with a birth incidence of 1 in 27,956 and 1 in 68,956, respectively (3). While typical cutaneous characteristics defined NF1 from other variants, hearing loss with vestibular dysfunction and severe pain distinguished NF2 and SWN, respectively (1, 4–6).

Neurofibroma, a kind of nerve sheath tumor that may grow close to the spinal cord, peripheral nerves, or cranial nerves, is characteristic of NF1. In addition to the pigmentary abnormalities that are usually present, it is possible to see dysplasia of the skeleton, low-grade gliomas, and involvement of many organ systems. Additionally, Eric Legius et al. provide the updated neurofibromatosis type 1 criteria in 2021 (7) (Table 1). The NF1 disorder progresses gradually during an individual's lifetime; however, the particular symptoms, the pace of advancement, and the severity of consequences significantly differ from person to person. Currently, there is no definitive treatment, and most clinical care is limited to monitoring and treating symptoms, most often through surgery. NF1 is caused by the NF1 gene, which codes for neurofibromin. This gene was found in 1990, and its function and significance in tumor formation and other NF1 symptoms have since been extensively studied. As a consequence of more excellent knowledge of NF1 clinical features, several targeted medications have emerged and are currently being explored in preclinical models and phase II clinical studies. This is an exciting time for NF1 patients, as new medicines on the horizon promise to improve their quality of life (QOL) (8).

**Table 1:** Revised diagnostic criteria for neurofibromatosis type 1 (NF1).

**A: The diagnostic criteria for NF1 are met in an individual who does not have a parent diagnosed with NF1 if two or more of the following are present:**
• Six or more café-au-lait macules over 5 mm in greatest diameter in prepubertal individuals and over 15 mm in greatest diameter in postpubertal individuals. • Freckling in the axillary or inguinal region. • Two or more neurofibromas of any type or one plexiform neurofibroma. • Optic pathway glioma. • Two or more iris Lisch nodules identified by slit-lamp examination or two or more choroidal abnormalities (CAs)—defined as bright, patchy nodules imaged by optical coherence tomography (OCT)/near-infrared reflectance (NIR) imaging. • A distinctive osseous lesion such as sphenoid dysplasia, b anterolateral bowing of the tibia, or pseudarthrosis of a long bone. • A heterozygous pathogenic NF1 variant with a variant allele fraction of 50% in apparently normal tissue such as white blood cells.
**B: A child of a parent who meets the diagnostic criteria specified in A merits a diagnosis of NF1 if one or more of the criteria in A are present**
a. If only café-au-lait macules and freckling are present, the diagnosis is most likely NF1 but exceptionally the person might have another diagnosis such as Legius syndrome. At least one of the two pigmentary findings (café-au-lait macules or freckling) should be bilateral. b. Sphenoid wing dysplasia is not a separate criterion in the case of an ipsilateral orbital plexiform neurofibroma.

However, the best treatment for neurofibroma and schwannoma is still the complete removal of the mass and capsule without causing injury to the attached organs. Recent advancements in minimally invasive surgery have led to the publishing of several different laparoscopic methods for treating retroperitoneal schwannomas. Unlike the reported cases of coincident GIST in NF1 patients that were managed regularly through a transperitoneal approach, either open or laparoscopically (9, 10). In our case, we did minimal invasive retroperitoneal laparoscopic surgery for duodenal GIST, which went as smoothly as usual for partial and total nephrectomy.

## Case report

**Figure 1:**
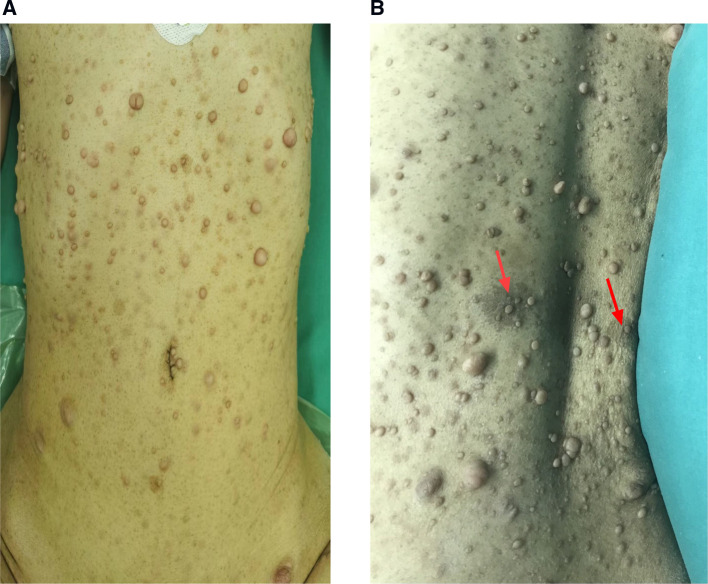
Individuals who have neurofibromatosis type 1 NF1 may exhibit a variety of cutaneous characteristics, including (**A**), the growth of nerve sheath tumors (neurofibromas)is a prominent hallmark of NF1. Neurofibromas can develop as isolated nodules or as cutaneous neurofibromas (**B**). pigmentary feature of NF1 patient (café-au-lait macules) on the back of the patient as (arrow).

**Figure 2:**
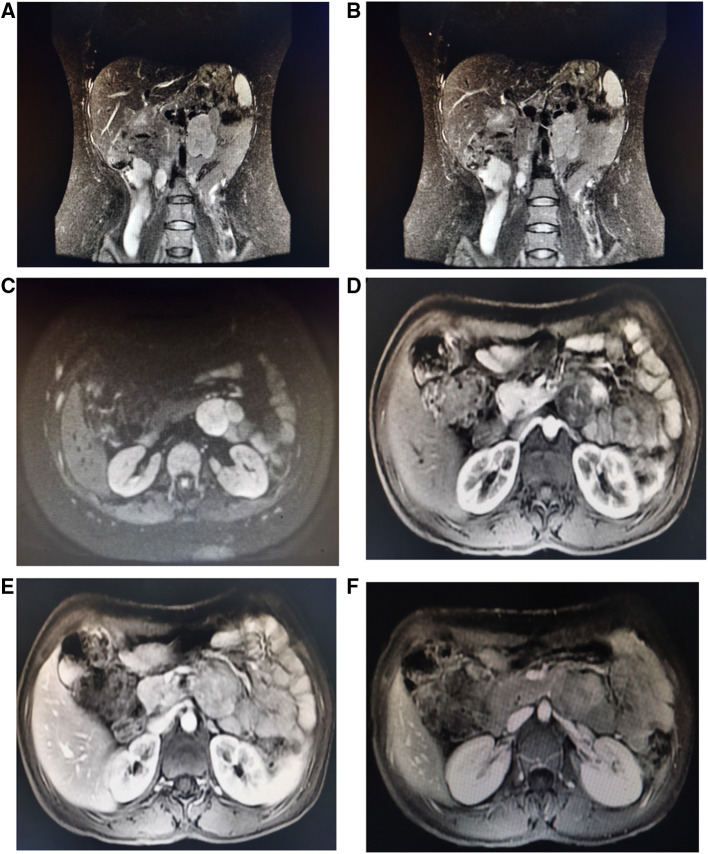
Abdominal MRI shows an irregular retroperitoneal mass under the pancreatic body and the pancreatic uncinate process is pushed forward to the right. The mass had about 3.8 cm × 5.7 cm × 2.8 cm in size, which has unclear boundary with some surrounding intestinal loops (**A,B**). Coronal T2-weighted image depicts high signal tumor; (**C**) Transverse Diffusion-weighted image shows obvious high signal tumor; (**D–F**) enhancement MRI scan on arterial, venous and excretion stages show uneven and obvious enhancement.

A 42-year-old female patient with neurofibromatosis was hospitalized at the department of general surgery with 10 days of right upper abdomen pain but no other symptoms such as diarrhea, vomiting, or bleeding. In addition, the patient said that she has had several nodules on her body for as long as she can remember and that both her mother and daughter have neurofibromatosis. However, during her abdominal MRI examination, a mass on the left side was found, which was described as a retroperitoneal tumor; she was then transferred to the department of urology. At the initial examination, significant café au lait spots and freckling were found over the patient's body, but the mass filling the left abdominal quadrant was inaccessible (Figure 1). Her blood pressure was 125/76 mmHg, and her heart rate was 74 beats per minute. Biochemistry and hematological tests revealed mild anemia (Hb: 97 g/L and Htc: 31.3 L/L); however, other parameters (liver function, renal function, electrolytes, coagulation function, blood cortisol, ACTH, blood aldosterone, renin, CRP, gastrin, insulin, and glucagon) were normal. No lesion or abnormality was discovered during the thorax CT scan evaluation. Abdominal magnetic resonance imaging (MRI) revealed an uneven tumor underneath the pancreatic body that occupies the left retroperitoneal region. The mass had about 3.8 cm × 5.7 cm × 2.8 cm in size, which has an unclear boundary with some surrounding intestinal tubes. The uncinate process in the pancreas is moved to the right. While the focus T1W image illustrates a low signal mass, the T2-weighted image indicates a high-signal tumor; and the diffusion-weighted displays an obvious high-signal tumor; additionally, enhanced MRI scans of the arterial, venous, and excretion phases clearly show uneven and noticeable enhancement (Figure 2). There were no gastrointestinal problems in our case. as well as the tumor's location based on imaging results led to the establishment of a primary diagnosis of retroperitoneal neurofibroma.

**Figure 3:**
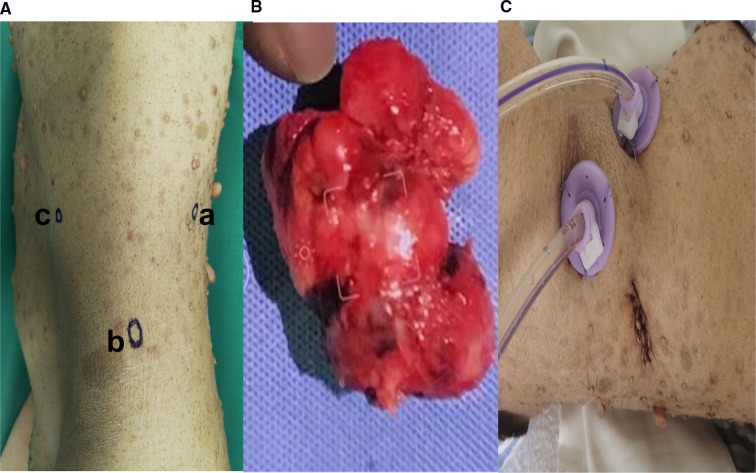
(**A**) Trocars configuration for left retroperitoneal laparoscopic approach; [trocar a] is inserted *via* 2 cm skin incision made below the 12th rib, anterior to the Sacro-spinal muscle, on the posterior axillary line and the skin incision is sutured to fix the trocar, [trocar b] A 10 mm camera trocar is inserted two fingers breadths above the iliac crest on midaxillary line; [trocar c] is inserted at the subcostal margin on anterior axillary line (**B**). Extracted tumor mass specimen (**C**). Postoperative drain in retroperitoneal space.

After three days of pre-operative preparation, the patient was taken to the operating room. Minimally invasive surgery was performed using a three-port retroperitoneal approach (Figure 3). A 2-centimeter incision is made on the posterior axillary line, beneath the 12th rib, anterior to the sacrospinal muscle. The muscle layer and lumbodorsal fascia were divided bluntly with a long hemostatic forcep. By insertion of the index finger into the retroperitoneal space (posterior pararenal space) and dissecting the fatty tissue from top to bottom and back to front, while simultaneously pushing the peritoneum anteriorly. Following that, the space is enlarged using a balloon expander. Subsequent trocar insertions will be directed from the retroperitoneal area using the index finger. Trocar 1 is entered *via* the first skin incision and sutured to secure the trocar, then a 10 mm camera trocar (Trocar 2) is placed two fingers breadths superior to the iliac crest on the midaxillary line; finally, trocar 3 is introduced on the anterior axillary line at the subcostal margin. On the dominant hand side of the surgeon, a 12 mm trocar is usually used, and a 5 mm trocar by a non-dominant one. CO2 insufflation *via* camera trocar with a pressure range from 10 to 14 mmHg to creates pneumoperitoneum. while the surgeon performs an operation on the patient's abdominal wall using trocar 1 and 3, the assistant stands on the backside holding camera using trocar 2. Firstly, retroperitoneal adipose tissue is mobilized from the infra-phrenic superiorly to the iliac fossa inferiorly and from the peritoneal reflection internally to the psoas major externally. after exposing the lateral conical fascia, it is longitudinally incised posterior to the retroperitoneal fold. After that, the dissection is conducted posteriorly between the posterior renal fascia and the psoas major, outside fascia. Due to the fact that the renal fascia is connected with quadratus lumborum fascia, these two fascias are always dissected together to expose the deeper psoas muscle fibers. superiorly, the plane of dissection extends to the diaphragm, while inferiorly it extends to the iliac fossa. Then, between the fusion fascia (the fascia posterior to the mesocolon) and the anterior renal fascia on the inferomedial pole of the kidney, precise anterior dissection is performed, accessing the first avascular plane (anterior pararenal space). At this point mass was seen and tumor boundaries dissection progress and we discovered a mass attached anteriorly to the duodenojejunal flexure. A portion of the duodenum was resected to remove the tumor mass, and primary anastomosis was accomplished through the retroperitoneal without manipulation of other abdominal organs.

**Figure 4:**
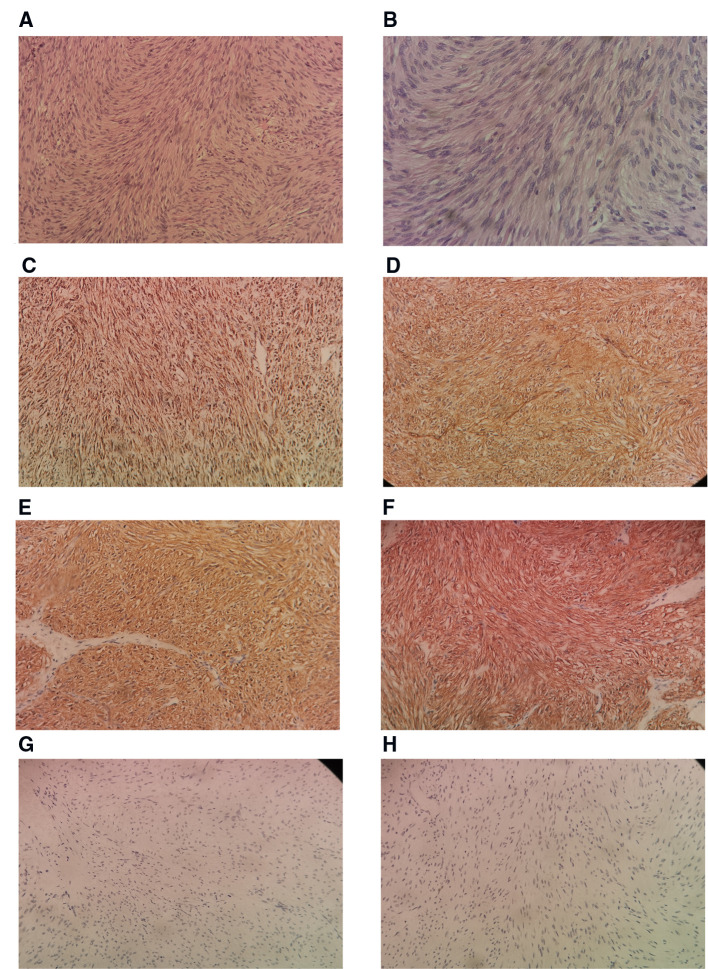
Histological sample. (**A**) Low power H & E Showing spindle shaped tumor cells arranged in fascicles and bundles. for tumor (**B**). Higher power view showing the spindle appearance of tumor cells. IHC positivity for SDH-B (**D**). IHC positivity for CD 34 (**E**). IHC positivity for DOG1 (**F**). IHC positivity for CD117 (**G**). IHC negativity for desmin (**H**). IHC negativity for S-100. H & E, hematoxylin and eosin stain; IHC, immunohistochemistry of tumor cells (200×).

A postoperative drain was placed through a small hole in the left posterior peritoneum, and a tumor mass of 6.5 cm × 5 cm × 3 cm in size was extracted. Following surgery, the patient spent one day in a critical care unit before being discharged to the wards. Finally, the drain was also put through the retroperitoneum to avoid peritonitis and reduce the risk of death, which is another advantage. On surgical day 9, the patient began an oral diet, and she was released from the hospital on day 14. Histopathologic investigations revealed that the resected mass was a composite of that CD117(+), CD34(+), desmin(−), DOG-1(+), Ki-67(+5%), S-100(−), SDH-B(+), and SMA(−), all of which are consistent with GIST tumor (Figure 4).

## Discussion

Neurofibromatosis type 1 is an uncommon neurogenetic condition characterized by pigmentary abnormalities, learning and social difficulties, and a susceptibility for benign and malignant tumor growth due to NF1 gene germline mutations (11). In comparison to other neoplasms, patients with NF1 had considerably lower disease-specific survival (DSS) rates if they developed undifferentiated pleomorphic sarcoma (UPS), high- grade glioma (HGG), malignant peripheral nerve tumor (MPNST), ovarian cancer, or melanoma. Individuals with NF1 have a significantly increased risk of developing a variety of neoplasms other than neurofibromas. Several of these are known to be associated with NF1, whereas others were previously unrelated to NF1. These neoplasms had significant associations with patient outcomes (5, 12). Life expectancy is 10 to 15 years less than in the general population, a decrease associated with malignant neoplasms (13).

Patients who have NF1 are born with mutations in just one allele of the gene that controls the tumor suppressor gene (Neurofibromin). The NF1 gene was cloned in the year 1990, and subsequent cell biology research has shown that neurofibromin, the product of the NF1 gene, mainly roles as a GTPase-activating protein (GAP) that hinders the RAS/MAPK pathway by increasing the hydrolysis of RAS-linked GTP. Recent developments in cell biology and animal models have led to the discovery of MEK antagonists as prospective treatment agents for plexiform neurofibromas (7, 14). A range of regionally and temporally distinct malignancies and other clinical manifestations is formed throughout development as a result of the loss of heterozygozygosity (LOH) of the other NF1 gene. These tumors and other clinical features are formed dependent on the cell type that is impacted. Over the past few years, a substantial amount of work has been put into tracing the origins of cancerous cells. This revelation has an important impact on both our understanding of underlying biology and our capacity to administer treatment that is specifically focused. In order to create an accurate model of how disease begins and progresses, it is necessary to discover where a tumor cell came from. In addition to this, it makes it possible to identify the molecular components that, in a step-by-step approach, accelerate the course of human cancer. Once we have a solid understanding of these stages, we will be able to locate important targets within tumor cells. In the context of NF1, it will be tremendously important to understand the subsequent steps that lead from Nf1 LOH to the creation of neurofibromas. Currently, there is virtually little treatment available for neurofibroma in NF1 patients other than surgical excision. This disparity between our current understanding of neurofibroma biology and clinical results could be explained by the lack of a reliable preclinical model that accurately depicts the cause of NF1 disease (14).

Thus far, the only therapeutic choices for cutaneous NFs have been surgical removal, various laser treatments, and electrosurgical excision (15). Selumetinib was authorized by the US Food and Drug Administration in April 2020 for the treatment of children with NF1-related symptomatic plexiform neurofibroma (16). Nearly all NF1 patients develop neoplasms neurofibromas,and in epidemiological studies, such as those conducted in Finland, show an absolute lifetime risk of malignancy of around 55%–60%, which is 5%–15% higher than the general population, 40% risk and an absolute excess of 15%–20%, as well as a life expectancy that is 10–15 years shorter than the general population (2, 5, 12, 17–19). Neurofibromas and plexiform neurofibromas are two common neurogenic tumors found outside the central nervous system (20). Plexiform neurofibromas are histologically benign tumors of the peripheral nerve sheath that affect up to 50% of people with neurofibromatosis type 1 and can cause significant consequences (16, 21). Neurofibromas in persons with NF1 can occur anywhere in the body, and at least 40% of affected adults have neurofibromas internally, though most are not noticeable on physical examination (22).

Primary retroperitoneal tumors are an infrequent, heterogeneous category of tumors originating outside the major organs in the retroperitoneal space (23). CT and MRI findings in conjunction with the patient's medical history can narrow down the possible diagnoses and even portray a retroperitoneal mass accurately. The histopathological results of neurofibromas must be consistent with their imaging characteristics (24). Neurofibromas on a CT scan have a uniform density and round shape with obvious, smooth edges. The CT density is reported at 20–25 HU on plain pictures, mildly and homogenously enhancing after contrast material administration, with a CT density of 30–35 HU on contrast-enhanced images. At MR imaging, neurofibromas may have a target-like in appearance with distinct behavior in the central region than in the periphery. In T1-weighted images, the central portion of the tumor is slightly hyperintense compared to the peripheral part, whereas in T2-weighted images, the periphery of the mass appears hyperintense. The central part is of intermediate signal intensity on T2-weighted images and enhances after gadolinium injection (23). Furthermore, NF1 may be associated with retroperitoneal tumors, the most malignant of which is MPNST, which has a significant progression rate and the potential for metastasis. Retroperitoneal tumors are uncommon and may be discovered inadvertently during imaging examinations. A pathology diagnosis should be considered, and the patient should be closely monitored, as malignant retroperitoneal tumors, such as MPNST, are possible (25, 26).

NF1 has been associated with several conditions, such as MEN2 syndrome, hereditary breast tumor, and GIST (27–30). Numerous GIST coexisting with NF1 have been documented, although the precise risk of developing GIST in NF1 patients remains unknown. In one postmortem research of over 27,000 cases, 3/12 (25%) of patients with NF1 had numerous GISTs, but clinical investigations show that GISTs are seen in 5%–25% of NF1 individuals (31). A GIST was observed in one-third of NF1 patients in an autopsy series, and a published review article noted that more than half of GISTs in NF1 were discovered accidentally, compared to just one in five individuals without NF1 (31–33). GIST is a rare mesenchymal tumor that almost always develops in the abdomen, specifically in the stomach or the small intestine. Symptoms may include abdominal discomfort, nausea, vomiting, bowel habits changes, or gastrointestinal tract bleeding (33). Many studies have been done on GISTs' morphological and immunophenotypic characteristics; the majority of CD117 and CD34-positive cells are robustly and diffusely stained, whereas desmin and S-100 protein are usually negative (31, 32). Surgical resection is a possibility for patients with GIST who have localized lesions, and neoadjuvant therapy with tyrosine kinase inhibitors is an option for those with advanced disease (33–36). Alterations in the KIT gene or particular platelet-derived growth factor receptor alpha (PDGFRA) gene aberrations in the cancerous cells indicate a positive response to this medication. On the other hand, cancers that lack KIT or PDGFRA mutations (“wild-type” GISTs) are often unresponsive to such therapy. Imatinib is not very effective for treating advanced GIST individuals who also have NF1 since the NF1 mutation seems to be the primary driver of the disease. As a result, the consensus is to avoid providing adjuvant imatinib to patients with NF1-related GIST unless an imatinib-sensitive mutation (e.g., KIT exon 11) is also present, which has rarely been described (33, 35, 37). While immunotherapies are approved in multiple cancer types, their role in the treatment paradigm of GIST is still unclear (38, 39). A review of studies of GIST in NF1 across databases for the last 10 years is identified in Table 2 (10, 40–48).

**Table 2:** List of reported cases of gastrointestinal stromal tumor in NF1.

Authors (ref.)	Year	Country	Sex/age (years)	GIST location	Presenting symptoms	Size	Associated condition	Management
Mishra A et al. (40)	2021	Nepal	Male, 57	Jejunum	Vomiting, melena	10.1 cm × 7.33 cm × 6.2 cm		Exploratory laparotomy
Arif AA et al. (41)	2021	Canada	Female, 67	Small-bowel	Abdominal pain and pneumoperitoneum	1 cm	Pancreatic Gastrinoma, Pheochromocytoma, and Hürthle Cell Neoplasm	Open surgery
Naoki Makita et al. (42)	2021	Japan	Female, 45	Duodenum	Fecal occult blood	4 cm	Neuroendocrine tumor	Open Pancreaticoduodenectomy
Tim N Beck et al. (43)	2020	USA	Male, 61	Distal jejunum	Hypertension, esophagitis and intermittent gastrointestinal bleeding	7 cm		Exploratory laparotomy
Park EK et al. (44)	2019	Korea	Female, 37	Proximal jejunum	Postprandial epigastric pain	1.5 cm and 1.6 cm		Pylorous- preserving pancreatoduodenectomy followed by adjuvant chemotherapy, consisting of etoposide and cisplatin
Park EK et al. (44)	2019	Korea	Male, 55	Duodenal 2nd portio	Incidentally	3 cm × 3 cm		Pancreatoduodenectomy
Park EK et al. (44)	2019	Korea	Female, 80	Retroduodenal mass	General weakness and weight loss	1.5 cm and 1.7 cm		Transduodenal ampullectomy and separate tumorectomy
Karolina Poredska et al. (45)	2019		Male, 58	Multiple GIST at proximal duodenum and jejunum	Hypertension, dyspepsia	(5–7 mm)	Right Pheochromocytoma	Surgery(a pancreaticoduodenectomy) Approach not mentioned
Dongfeng Pan et al. (46)	2016	China	Male, 56	Small intestine	Hypertension, abdominal pain	(1.3 cm × 1.3 cm × 1 cm)	Left Pheochromocytoma	Surgery excision
Hakozaki Y et al. (10)	2017	japan	Female, 70	Duodenum	positive fecal occult blood	6 mm nodule	Rectal carcinom	Laparoscopic anterior resection
Myrella Vlenterie et al. (47)	2013	Netherlands	Female, 59	Stomach and small intestine	extreme fatigue	3 cm and 0.8 cm in diameter	Left adrenal gland	Open surgical removal
Myrella Vlenterie et al. (47)	2013	Netherlands	Male, 55	Jejunum	Hypertension and tachycardia	4 mm	Bilateral adrenal gland	Open abdominal exploration and tumor resection
Beyza Ozcinar et al. (48)	2013	Turkey	Male, 48	Small intestine	Hypertension And melena	(1.5–3.5 cm)	Right adrenal gland	Transabdominal approach with tumor resection
Present study	2022	China	Female, 42	Duodenum		6.5 cm × 5 cm × 3 cm		Laparoscopic retroperitoneal resection

Despite advances, surgical excision remains the standard of care for non-metastatic GIST (24, 40, 41). The majority of GIST cases in neurofibromatosis type 1 have been described using an open technique, primarily exploratory laparotomy. The development of minimally invasive surgery has impacted the number of procedures performed. Surgery using the laparoscopic approach is rapidly becoming the standard of care for numerous operations due to the generalized benefits of lower pain, shorter hospital stays, and speedier return to regular life activity. The use of minimally invasive surgery in oncologic procedures is a point of dispute. A significant amount of research has been conducted on a number of different cancers to demonstrate that a laparoscopic technique can be safe and result in a safe oncologic margin. Additionally, research has been conducted to determine whether or not a laparoscopic method is as effective as open surgery and results in comparable oncologic outcomes. There has been limited consensus on the use of minimally invasive methods in the excision of GISTs since GISTs are a newly identified entity and a rare neoplasm. This lack of agreement is mostly due to the fact that GISTs are uncommon. The surgical therapy of GIST has undergone a significant shift ever since Lukaszczyk and Preletz reported the first laparoscopic removal of a stomach GIST identified unintentionally during a cholecystectomy (9, 49). Previous researchers have looked into laparoscopy as a potential treatment for GIST removal. The biological properties of these tumors make laparoscopic resection a preferable treatment option for removing them, despite the fact that there is not yet a widespread agreement about the significance of minimally invasive techniques in their removal. Local excision rather than formal organ resection has been the preferred treatment strategy for GISTs as a result of the rarity of submucosal and lymphatic invasion. This has made laparoscopic resection an enticing alternative to traditional surgery, which is more intrusive. Large resection margins have always been recommended, despite the fact that there has been no relationship established between them and enhanced survival or recurrence. As a direct consequence of this, extensive margins and the dissection of lymph nodes are not required. It is generally agreed upon that achieving a negative gross surgical margin is essential for lowering the risk of GISTs returning locally and spreading to other organs. It was advised that laparoscopy be reserved for GISTs that are smaller than 2 centimeters in size. This recommendation was made due to concerns regarding tumor rupture and seeding of the peritoneum and the capacity to construct an appropriate oncologic margin. In spite of these challenges, surgeons continued to resect GISTs laparoscopically with success, prompting the NCCN to alter the criteria contained in their 2010 Task Force Report to include GISTs measuring up to 5 centimeters as candidates for laparoscopic resection (49). GIST resection surgery is governed by the ideas of retaining an intact capsule to avoid tumor spillage and establishing a negative margin to secure thorough excision of localized illness. These principles help ensure that all diseased tissue is removed from the body during the procedure.

A retroperitoneal tumor can be in close contact with structures such as the duodenum, renal vein, and IVC, which often require meticulous dissection to avoid Damage, like in our case in which the final diagnosis was GIST of the duodenum that originated from its stroma. Second, with recent advances in the field of minimally invasive surgery, several laparoscopic approaches to retroperitoneal schwannomas have been reported (50–52). Laparoscopic surgery, which has become a useful and feasible option for this procedure, is associated with minimal invasiveness and early postoperative recovery (50). We have been done the duodenal stromal tumor retroperitoneal laparoscopy and avoided open surgery or entering the abdomen. Retroperitoneal laparoscopic surgery for duodenal tumors is rare, but our surgery went smooth, and the patient recovered quickly because we did not enter through the abdominal cavity and had little manipulation of other intestines.

## Conclusion

A histological diagnosis should be deemed required since it is possible to misdiagnose a retroperitoneal neurofibroma as another kind of tumor, such as a GIST that is linked with individuals who have NF1. When dealing with retroperitoneal structures, the laparoscopic retroperitoneal method is one that is not only almost risk-free but also offers a number of benefits. Anatomically challenging locations, such as the duodenal GIST in NF1 individuals, can be managed effectively by using the minimally invasive laparoscopic retroperitoneal approach even when retroperitoneal neoplasia arises from the intrabdominal structures. For the purpose of demonstrating the optimum safety and efficacy of this method, more large cohort studies need to be carried out.

## Data Availability

The original contributions presented in the study are included in the article/Supplementary Material, further inquiries can be directed to the corresponding author/s.
